# Illustrating phallic worship: uses of material objects and the production of sexual knowledge in eighteenth-century antiquarianism and early twentieth-century sexual science

**DOI:** 10.1080/02666286.2017.1294952

**Published:** 2017-07-03

**Authors:** Jana Funke, Kate Fisher, Jen Grove, Rebecca Langlands

**Keywords:** sexuality, sexual science/sexology, antiquarianism, material culture, Richard Payne Knight, Magnus Hirschfeld

## Abstract

This article reveals previously overlooked connections between eighteenth-century antiquarianism and early twentieth-century sexual science by presenting a comparative reading of two illustrated books: *An Account of the Remains of the Worship of Priapus*, by British antiquarian scholar Richard Payne Knight (1750–1824), and *Die Weltreise eines Sexualforschers* (The World Journey of a Sexologist), by German sexual scientist Magnus Hirschfeld (1868–1935). A close analysis of these publications demonstrates the special status of material artefacts and the strategic engagement with visual evidence in antiquarian and scientific writings about sex. Through its exploration of the similarities between antiquarian and sexual scientific thought, the article demonstrates the centrality of material culture to the production of sexual knowledge in the Western world. It also opens up new perspectives on Western intellectual history and on the intellectual origins of sexual science. While previous scholarship has traced the beginnings of sexual science back to nineteenth-century medical disciplines, this article shows that sexual scientists drew upon different forms of evidence and varied methodologies to produce sexual knowledge and secure scientific authority. As such, sexual science needs to be understood as a field with diverse intellectual roots that can be traced back (at least) to the eighteenth century.

## Introduction

This article reveals hitherto overlooked connections between eighteenth-century antiquarianism and early twentieth-century sexual science by offering a comparative reading of two illustrated books: *An Account of the Remains of the Worship of Priapus*, by British antiquarian scholar Richard Payne Knight (1750–1824), and *Die Weltreise eines Sexualforschers* (The World Journey of a Sexologist), by German sexual scientist Magnus Hirschfeld (1868–1935). The use of illustrations in these publications shows that both authors drew upon historical artefacts as evidence and points to the special status of material objects within antiquarian and scientific writings about sex. Considering in tandem Knight’s and Hirschfeld’s engagement with material objects, the article demonstrates the centrality of material culture to the production of sexual knowledge in the Western world. Through its exploration of the relationship between antiquarian and sexual scientific thought, it opens up new perspectives on the intellectual origins of Western sexual science.

Knight’s long essay on phallic rituals and cults, published as part of his *Worship of Priapus*, is based chiefly on Greek and Roman antiquities, especially the phallic material newly discovered in the excavations at Pompeii and Herculaneum.^1^ Knight brings these objects into dialogue with contemporary wax phalluses found in Italy to articulate an argument about the universal origins of religion in the worship of sex. Working 150 years later, Hirschfeld, like other sexual scientists of his time, sought to study sex by drawing on diverse forms of knowledge and expertise.^2^ The *World Journey* presents the travel narrative he wrote while travelling through America, Japan, China, Taiwan, the Philippines, India, Egypt, and Palestine between November 1930 and April 1932. It demonstrates Hirschfeld’s interest in viewing sex from cross-cultural and cross-historical perspectives. In it, he provides examples of ritual phallic objects in Japan, Indonesia, and India to illustrate the prevalence of phallic worship within Asian cultures, and to support his claims about the unrecognized importance of ritual practices relating to sex across human societies.^3^

Knight and Hirschfeld both collected phallic objects and make frequent reference to phallic artefacts in *Worship of Priapus* and *World Journey*.^4^ As this article shows, the engagement with material culture in these works fulfils three interrelated functions. Knight and Hirschfeld draw attention to the uncertainties involved in understanding material remains which have held different meaning in different historical and cultural contexts. Both authors highlight and exploit this ambiguity of material objects to raise questions about how different cultures across history have understood sex, and to draw attention to the historical contingency of Western sexual attitudes. In this sense, the engagement with material culture is central to Knight’s and Hirschfeld’s comparative cross-cultural and cross-historical approach to sexual knowledge. In addition, both authors emphasize the hidden meaning of phallic objects, which are often associated with religious beliefs. Knight and Hirschfeld demonstrate their scholarly expertise by recontextualizing the artefacts in their past, and by revealing what they present as the objects’ original meaning. In this way, material objects facilitate the construction of both authors’ authority over the subject matter. Moreover, material artefacts are seen to possess an immediacy that creates a sense of connection with the past and speaks straightforwardly to later viewers. Knight and Hirschfeld make use of this particular appeal of material remains and suggest that historical phallic objects provide evidence of cultures that were less restrictive in their sexual attitudes than the contemporary Western world. In so doing, both authors draw upon material objects to expose and challenge what they perceived as restrictive Western attitudes towards sex.

The use of visual illustrations of phallic objects in *Worship of Priapus* and *World Journey* is key to the presentation of these arguments in both volumes. Knight had commissioned the engraver James Newton (1748–1804) to produce illustrations of phallic objects, many of which were in Knight’s own collection or those of his friends, and eighteen plates were included in the first edition of *Worship of Priapus*.^5^*World Journey* presents a long written account of Hirschfeld’s travels together with forty-seven photographs.^6^ Ten of these images depict artefacts or sites related to phallic worship.^7^ Both works draw strategically on culturally entrenched and historically specific ideas about the differences between word and image to complement and enhance Knight’s and Hirschfeld’s textual engagement with material culture. The illustrations ground both authors’ scholarship in empirical data and are central to their comparative research method. In addition, the images draw attention to the challenging task of interpreting historical remains, thus showcasing both authors’ scholarly expertise. Finally, the illustrations are used to draw upon the specific appeal of material objects: the visual representations offer phallic artefacts up to the perusal of readers and are seen to allow immediate access to the past. Both Knight and Hirschfeld point out that the illustrations are valuable precisely because visual images are more explicit in their depiction of sex than textual descriptions. As such, illustrations are seen to play a particular role in exposing and breaking with allegedly restrictive Western attitudes.

While antiquarianism and sexual science need to be understood as products of distinct historical moments, Knight’s and Hirschfeld’s shared interest in material culture and their similar use of illustrations expose striking continuities between antiquarian and sexual scientific thought. In developing these insights, this article presents alternative perspectives on Western intellectual cultures that have particular implications with regard to entrenched historiographical understandings of the emergence of sexual science. Western sexual science has frequently been seen as a field that develops in the mid- to late nineteenth century and is rooted primarily in medical disciplines like forensics, neurology, and psychiatry.^8^ In keeping with this predominantly medical view of sexual science, scholarship has tended to focus on the patient case study as the key form of evidence favoured by sexual scientists.^9^ This article challenges such narratives of the emergence of sexual science in two ways. First, it shows that, in addition to the patient case study, sexual scientists made frequent use of anthropological and archaeological evidence. The cross-cultural and cross-historical thinking inspired by the engagement with historical objects and their illustrations played an important role in producing sexual knowledge and securing scientific authority. Second, by investigating the intellectual traditions underpinning such cross-historical and cross-cultural thinking about sex through the example of phallic worship, this article reveals previously overlooked connections between early twentieth-century sexual science and eighteenth-century antiquarianism. As such, it opens up a new understanding of a longer and more diverse intellectual history of Western sexual science.

## Antiquarianism, sexual science, and phallic worship

*Worship of Priapus*, first published in 1786, is firmly located within intellectual traditions of seventeenth- and eighteenth-century antiquarianism in which the collection and empirical study of material remains, especially those of antiquity, took centre stage.^10^ The work was published at a point when antiquarian scholarship was being transformed following the excavations of Pompeii and Herculaneum in the early and mid-eighteenth century. The scale and richness of the finds sparked interest in the collection and study of antiquities, and Naples became the centre of a circle of antiquarian scholars. Many of the British contingent of antiquarians in Naples, including Knight, were members of the Society of Dilettanti, a gentleman’s club which fostered historical scholarship and the collection of ancient artefacts.^11^ The society supported Knight’s publication of *Worship of Priapus*, and its members were his intended readership: ‘I meant my discourse only for the Society and a few real dilettanti.’^12^ The publication is a key work in the intellectual development of antiquarian scholarship. Building on earlier antiquarian thought, Knight’s close reading of material objects as a means of understanding the past and especially his comparative use of artefacts became distinctive features of eighteenth-century antiquarianism^13^

Knight’s core thesis describes how the origins of all ancient religions can be located in the worship of ‘generative’ or ‘creative’ powers. The evidence for Knight’s argument comes from a sustained close engagement with imagery from ancient cultures, in particular the image of the phallus which was ubiquitous in ancient art and specifically the phallic material newly discovered in the excavations at Pompeii and Herculaneum.^14^ Knight also learnt of the recent Western discovery of erotic Hindu temple art and other Asian iconography, which inspired cross-cultural comparative scholarship.^15^ However, the debt to classical cultures is evidenced in the title of Knight’s cross-cultural survey: Priapus, the Greco-Roman fertility god, would become permanently associated not only with all Roman but also with *universal* fertility rites across cultures and historical periods.

Hirschfeld’s *World Journey* does not refer directly to *Worship of Priapus*, as discussed in the final section of this article, but is framed as a contribution to ‘sexual ethnology’ (Sexualethnologie), a sub-field connecting sexual science and anthropology.^16^ It is a key example of the ways in which the anthropological study of non-Western sexual behaviours and customs featured in early twentieth-century Western sexual science.^17^ Hirschfeld’s engagement with phallic worship focuses primarily on sexual reproduction and, specifically, female sexual behaviour and fertility. In contrast to some of his other publications, aimed primarily at medical or legal professionals, *World Journey* targeted a more diverse audience. It addressed readers with a professional interest in sexual science and anthropology, but was written in an accessible style and published without sexually explicit passages or images, thus also appealing to a broader readership.

While Hirschfeld’s focus on Asian rather than ancient Greek and Roman cultures distances him from Knight, his understanding of phallic worship closely parallels the arguments developed in *Worship of Priapus*. Like Knight, Hirschfeld was interested in the restrictive influence of religious thought on sexual practices and argued that many religions, including Christianity and Buddhism, endorsed an ascetic worldview that was opposed to phallic worship.^18^ Both Knight and Hirschfeld saw phallic worship as indicative of primitive sexual and fertility rites that were once found across cultures, but had since been subjected to censorship due to the rise of Western religious domination and other civilizing influences. Like Knight, Hirschfeld’s fascination with phallic worship was also tied to his interest in an undifferentiated archaic form of sexual desire that could encompass diverse sexualities.^19^

Hirschfeld’s engagement with phallic worship reflects the longer legacy of *Worship of Priapus*, and is indicative of the influence of Knight’s ideas on the interrelated fields of sexual science, anthropology, archaeology, comparative religion, and folklore studies from the mid-nineteenth century onwards.^20^ In Britain, the years around 1865 witnessed a significant revival of interest in Knight’s work. The Anthropological Society of London (ASL) published studies of phallic worship that drew explicitly on *Worship of Priapus*, and reprinted many of its illustrations.^21^ The reception of Knight’s ideas at this historical moment was shaped by nineteenth-century colonialism: cultures that had only figured briefly in *Worship of Priapus*, such as India, and others that Knight had not considered at all, for instance contemporary African societies, were now treated in more detail.^22^ In these studies, ‘carefully executed drawings’ of material culture, for example Japanese ‘phallic temples’, were prized as key evidence.^23^ In keeping with this trend, the ASL republished the *Worship of Priapus* in 1865, in a run of five hundred, adding their own illustrations to accompany a new essay on the ‘generative powers’ in medieval Europe.^24^ Many of these new images were of material from a ‘Collection Illustrative of Phallic Worship’, put together by one member of the ASL, former medical doctor and collector George Witt (1804–1869).^25^ This revival of interest in Knight’s work on phallic worship continued to influence European scholarship, including that of Hirschfeld and his contemporaries, in ways that have not yet been understood and examined fully.^26^

## Illustrations of phallic objects as empirical evidence

In addition to the direct influence of Knight’s ideas on early twentieth-century sexual science (often mediated through the nineteenth-century reception of *Worship of Priapus*), antiquarian thought also shaped sexual scientific thinking on a methodological level. Recent scholarship has begun to draw attention to the influence of antiquarian methodologies on the development of empirical methods in the human sciences, especially anthropology, ethnography, and archaeology, but also history.^27^ Tracing the significance of antiquarian legacies in nineteenth- and early twentieth-century intellectual culture demonstrates further the previously unexplored linkages between antiquarianism and sexual science. Knight’s and Hirschfeld’s engagement with phallic objects and their illustrations is indicative of a shared investment in comparative methodologies that served to produce sexual knowledge and affirm scientific authority.

Antiquarians like Knight drew on the language of scientific experimentation and saw their work as based on rigorous empirical observation rather than ‘theory’; they were explicit in condemning speculation, imaginative reconstruction, or hyperbole.^28^ Central to antiquarian thought was the collection and comparison of material objects from different historical periods and cultural contexts. Such antiquarian approaches were mocked as mere information gathering, especially within early nineteenth-century British debates about the purpose of studying the past, which often denigrated antiquarians as producing scholarship of no significance.^29^ Yet this dismissive rhetoric obscures the ways in which later methodologies for approaching the past and understanding other cultures continued to be informed by antiquarian thought, particularly when the human sciences increasingly sought to fashion themselves as ‘scientific’ disciplines later on in the nineteenth century.^30^ At this point, historians placed new emphasis on empiricism and the close analysis of evidence, which required the kind of observational detachment and close attention that antiquarians had previously championed.^31^ In addition, the comparative methodologies developed by antiquarian thinkers, together with their theories about the cultural evolution of ideas based on material culture, facilitated the development of anthropology and ethnography as disciplines.^32^ Nineteenth- and twentieth-century intellectual cultures were thus deeply indebted to antiquarian scholarship, even if they often self-consciously sought to invent new ‘modern’ approaches to studying the past and other cultures.

While *Worship of Priapus* predates the late nineteenth- and early twentieth-century emergence of sexual science as an explicitly demarcated field of knowledge, Knight anticipated later sexual scientific approaches in a number of ways: he authorized the presentation and discussion of sexually explicit material by appealing to the principles of dispassionate and rational observation, brought together ancient sources and ethnographic evidence, and pioneered the study of contemporary (folkloric or ‘primitive’) customs as a window onto past practices. In so doing, he set in train a comparative method that influenced approaches in the human sciences and shaped the emergence of a cross-disciplinary sexual science.

Underpinning Knight’s and Hirschfeld’s comparative engagement with phallic worship traditions was the desire to resist and challenge Western sexual attitudes. Knight’s work must be understood in the context of his anticlericalism and his active resistance to the Catholic establishment.^33^ In *Worship of Priapus*, he rejects how ‘the zealous propagators of the Christian faith’ have condemned phallic worship as ‘obscene’ rather than considering its ‘symbolic’ dimensions and ‘original meaning’.^34^ As he explains, phallic worship ‘will be found to be a very natural symbol of a very natural and philosophical system of religion, if considered according to its original use and intention’.^35^ Thus, Knight argues that unbiased cross-cultural and cross-historical comparison was key to the production of a more authoritative form of scholarship that moved beyond distorted views on sex.

Although it took shape in a different social and political context, there were similar tendencies within late nineteenth- and early twentieth-century sexual science to present sexual scientific thought as progressive and liberationist. Hirschfeld conducted his research under the motto ‘per scientiam ad justitiam’ (justice through science), which is indicative of his faith in modern science to replace biased views of sex, and drive social and political change.^36^ He fought against the criminalization of homosexuality and sought to offer evidence of his doctrine of *‘sexuelle Zwischenstufen’* (sexual intermediaries), which insisted on the variability of gender and sexual expression.^37^ To this end, *World Journey* exposes readers to the different ways in which gender and sex were understood, organized, and experienced in cultures around the world. In particular, Hirschfeld engages with phallic worship in Asian cultures to introduce his Western readership to an allegedly less restrictive experience of sexual desire that he can access by virtue of his scientific expertise.

The visual representations of phallic objects play a crucial role in supporting Knight’s and Hirschfeld’s arguments and providing material evidence of phallic worship. The use of illustrations in *Worship of Priapus* and *World Journey* is underpinned by the perception that images offer a more immediate form of representation and a superior kind of empirical knowledge.^38^ At the time of Knight’s writing, engravings were commonly used in antiquarian research.^39^ They were seen to be ‘documents of real objects’ and often perceived as more authentic in their depiction of the material world than other visual techniques and written descriptions.^40^ Knight was particularly outspoken about the significance of the often explicit engravings in *Worship of Priapus*. In a letter to fellow Dilettante Joseph Banks from 1785, he discusses the possibility of editing the book to make it suitable for a wider public readership beyond the circle of the Dilettanti Society. However, the plan faltered on the grounds that no bowdlerization could be performed on the engravings, which were deemed too important to be cut out:

I shall be happy in […] finding some expressions which may not give offence to the Godly, though I fear that it will be impossible to make the work fit for any but very profane persons on account of the prints which are necessary to explain it. […] Holy spirit may be changed into divine spirit [… but] if it is to be in the smallest degree public many other parts must be unpublished.^41^

The engravings were integral to *Worship of Priapus* because they provided crucial empirical evidence of phallic worship. They also facilitated Knight’s comparative typological approach to material culture. This method consisted of comparing different objects and pointing to shared features, in this case the symbolic sexual meaning of phallic artefacts, which allowed Knight to suggest that there were previously overlooked analogies between Christian and pre-Christian traditions.^42^ In fact, several of the engravings included in *Worship of Priapus* (e.g. figures 3 and 6) depict objects from different cultures side by side on the same page, thus reinforcing Knight’s thesis concerning the unexpected links between these artefacts, and inviting readers to participate in his comparative reading.

**Figure 1.**
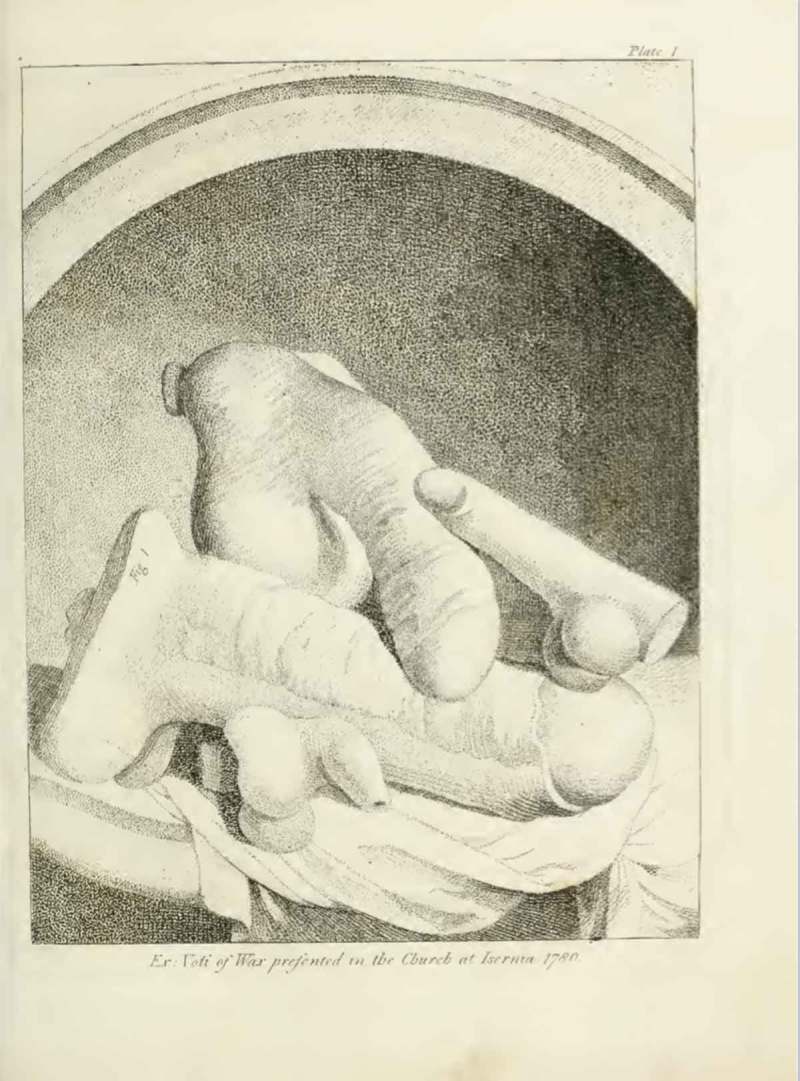
‘Ex voti of wax presented in the church of Isernia 1780’, i.e. votive wax phalluses; from Richard Payne Knight, *An Account of the Remains of the Worship of Priapus* (London: T. Spilsbury, 1786), pl. I.

**Figure 2.**
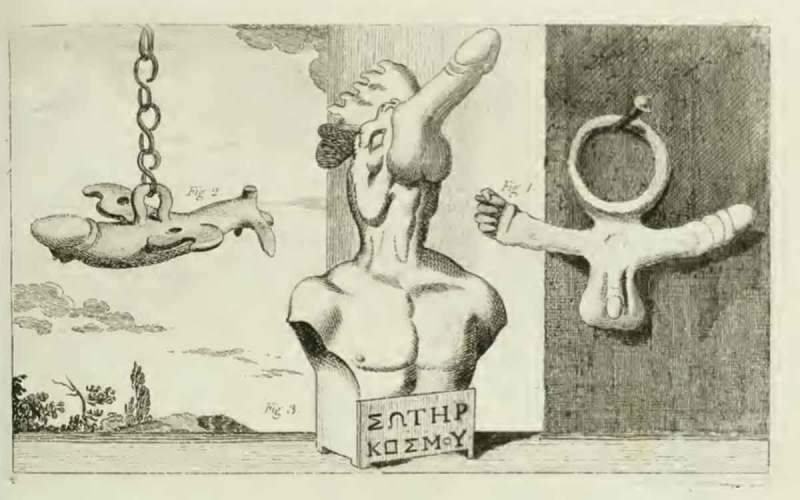
Ancient Roman amulets and (possibly modern) sculpture; from Richard Payne Knight, *An Account of the Remains of the Worship of Priapus* (London: T. Spilsbury, 1786), pl. II.

**Figure 3.**
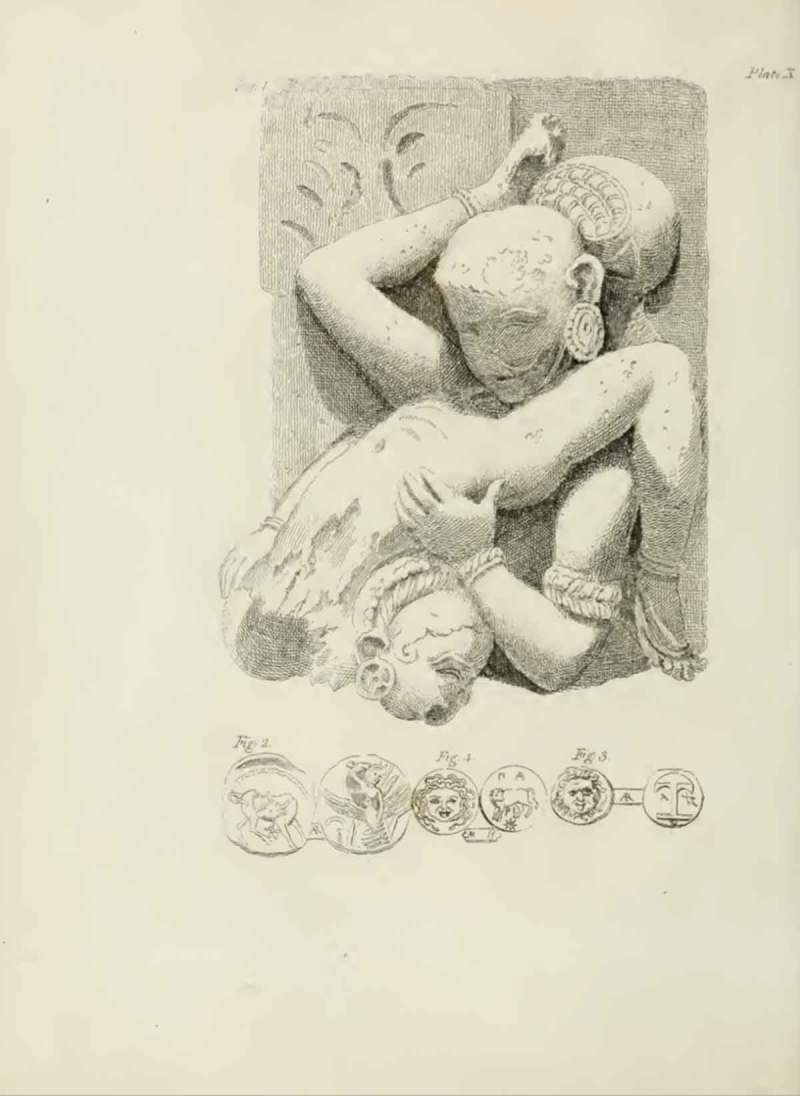
Carving from the Elephanta Caves, near Mumbai, India, together with Ancient Greek coins and medals; from Richard Payne Knight, *An Account of the Remains of the Worship of Priapus* (London: T. Spilsbury, 1786), pl. X.

**Figure 4.**
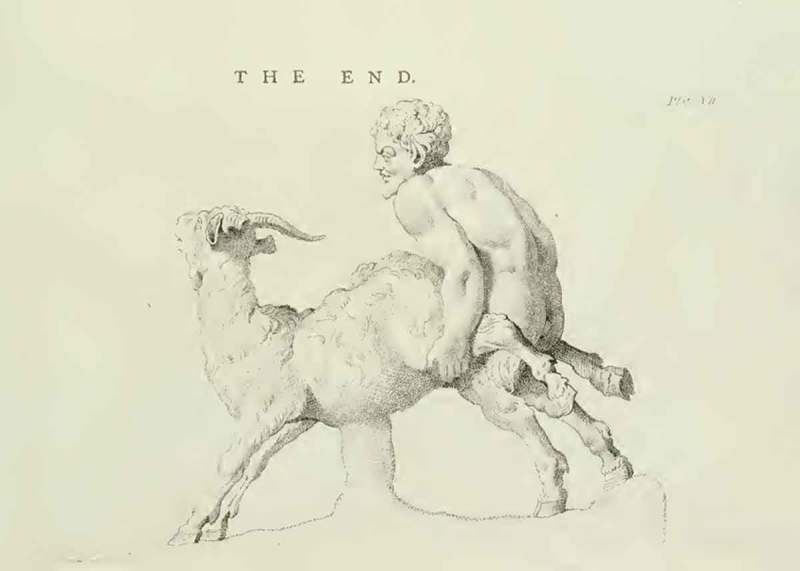
‘The end’, showing Pan and the Goat sculpture; from Richard Payne Knight, *An Account of the Remains of the Worship of Priapus* (London: T. Spilsbury, 1786), pl. VII.

**Figure 5.**
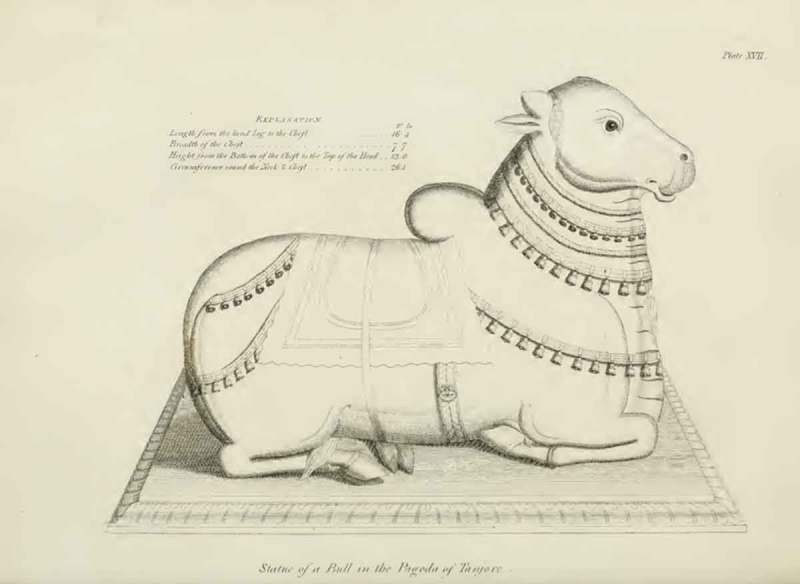
Statue of a bull in the Pagoda of Tanjore [Thanjavur]; from Richard Payne Knight, *An Account of the Remains of the Worship of Priapus* (London: T. Spilsbury, 1786), pl. XVII.

**Figure 6.**
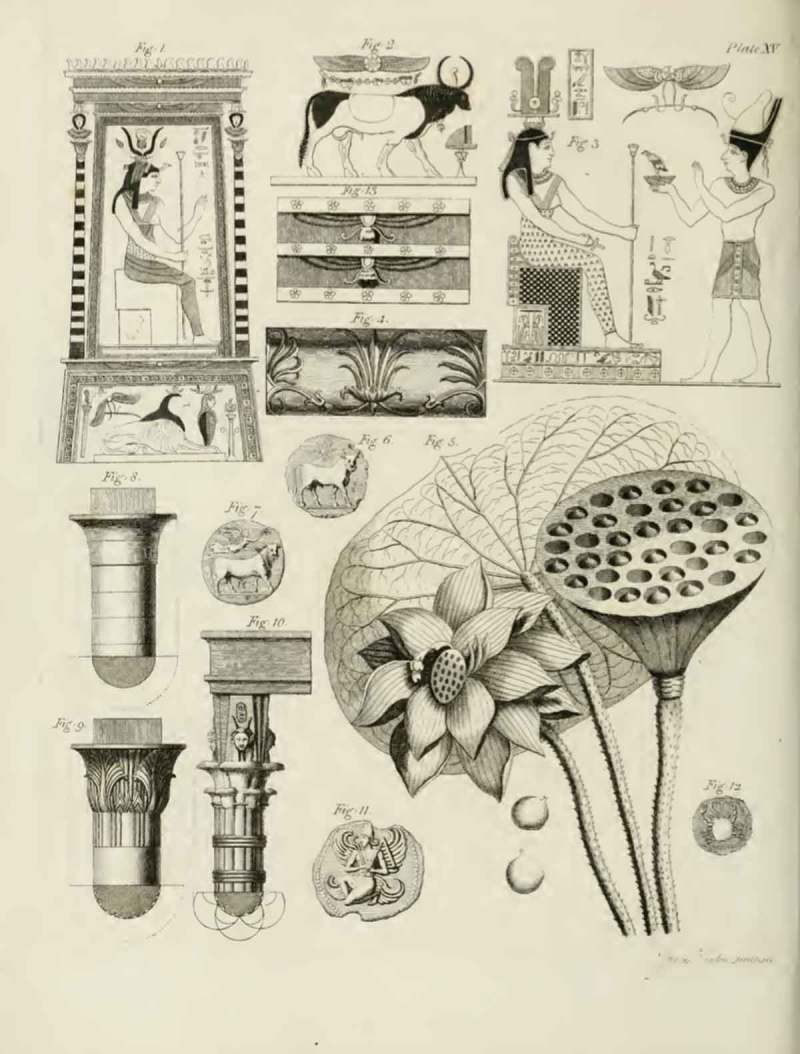
Cross-cultural objects, including images of lotus flowers from Ancient Egypt and Ancient Greece; from Richard Payne Knight, *An Account of the Remains of the Worship of Priapus* (London: T. Spilsbury, 1786), pl. XV.

Over the course of the nineteenth century, photography replaced earlier visual reproduction techniques and came to be seen as the most accurate way of depicting reality. By the mid-nineteenth century, photographs were increasingly used to record artefacts of antiquarian interest and took the place of engravings in new antiquarian publications and collections.^43^ They were also drawn upon to offer what was perceived as objective empirical evidence in disciplines seeking scientific authority.^44^ Hirschfeld made frequent use of medical photographs as part of his patient case studies, for example, in his *Sexualpathologie* (Sexual Pathology).^45^ He also included photographs in publications aimed towards more general audiences, such as the city guide *Berlins Drittes Geschlecht* (Berlin’s Third Sex). In all these publications, photographs depicting, for instance, homosexual, cross-dressing, or sadomasochistic individuals are used to provide empirical evidence of the infinite variability of gender expression and sexual desire.

**Figure 7.**
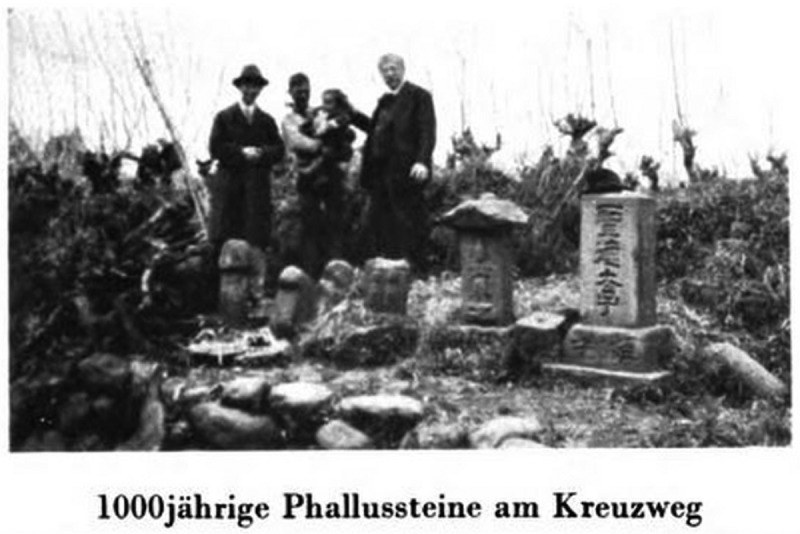
‘1000-year old phallic stones at the crossroad [Japan]’; from Magnus Hirschfeld, *Die Weltreise eines Sexualforschers* (Brugg: Bözberg, 1933), pl. 7.

**Figure 8.**
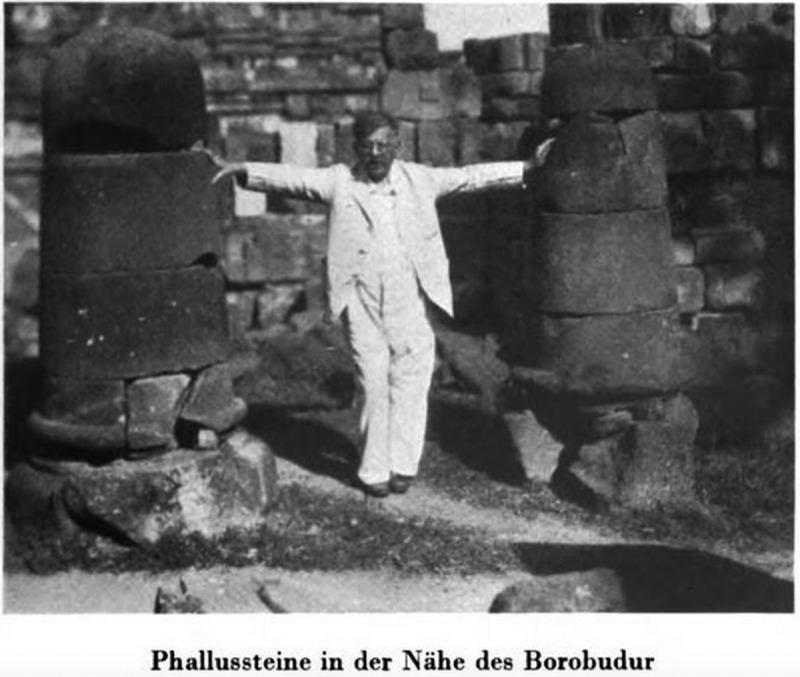
‘Phallic stones near Borobudur [Java]’; from Magnus Hirschfeld, *Die Weltreise eines Sexualforschers* (Brugg: Bözberg, 1933), pl. 19.

**Figure 9.**
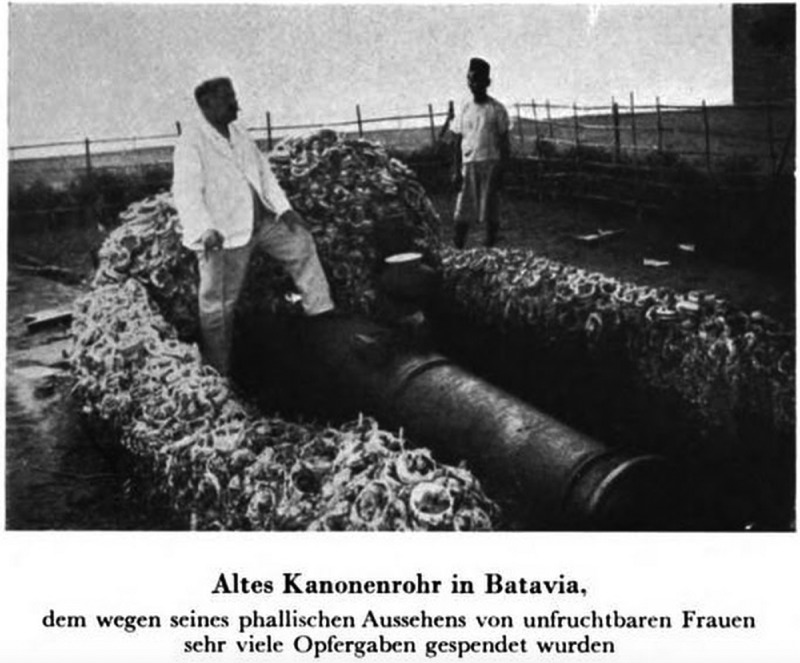
‘Old cannon in Batavia, whose phallic appearance inspires infertile women to make many sacrificial offerings [Java]’; from Magnus Hirschfeld, *Die Weltreise eines Sexualforschers* (Brugg: Bözberg, 1933), pl. 19.

The photographs included in *World Journey* serve a similar purpose by providing empirical evidence of the diversity of sexual practices across non-Western cultures. As such, they reflect the anthropological fascination with the photographic image as a means of capturing an allegedly authentic record of cultural difference.^46^ Drawing on photographs to record material objects, the volume also stands in the tradition of antiquarian uses of visual reproduction techniques. In contrast to those engravings in *Worship of Priapus* that depict several objects in the same image, Hirschfeld’s photographs show single objects or objects of the same kind, which are often presented in their original environment (figures 7–9). These photographic images draw attention to the singular qualities of the objects embedded in the unique cultural contexts of their creation. This reflects the understanding, shared by Hirschfeld and Knight, that it was imperative to study historical artefacts on their own terms and in an unbiased fashion. At the same time, *World Journey* also promotes a comparative reading, for instance, by grouping photographs of different phallic objects together and by using the textual framework to tease out connections between these artefacts.^47^ Like Knight, Hirschfeld thus uses illustrations to offer authoritative proof of the existence of phallic worship across historical and contemporary Asian cultures.

Despite the shared emphasis on the empirical value of images, both *Worship of Priapus* and *World Journey* betray anxieties about the second-hand nature of illustrations which are addressed in the textual discussion of these images. Knight emphasizes that he and his fellow antiquarians have personally seen many of the artefacts in question and that they are part of their own collections. Similarly, Hirschfeld authorizes his anthropological knowledge by stressing that he has personally handled, collected, and photographed phallic artefacts. His physical presence in three of the photographs (figures 7–9), which show him posing with different phallic objects, offers further evidence of his first-hand experience of the material remains and the cultures in which they originate. These textual passages not only seek to guarantee the authenticity of the illustrations but also offer another means through which Knight and Hirschfeld can affirm their expert knowledge, since it is evident that they have had privileged access to many of the material artefacts on which their arguments are based.

## The interpretation of phallic objects and scientific authority

If illustrations are seen to have a particular value in offering empirical evidence, Knight and Hirschfeld also emphasize that the engravings and photographs, just like the objects they depict, do not speak for themselves but require careful analysis and interpretation which is offered in the textual framework.^48^ While the ideas developed in writing are reliant on the visual representations of phallic artefacts, both authors emphasize that the textual framework is needed to illuminate the meaning of the illustrations and the material objects they depict. The layout of both volumes reflects this mutually dependent relationship of word and image. Knight places the illustrations throughout *Worship of Priapus*, thus creating dialogue between the textual descriptions and the images. He also uses footnotes to refer his readers to the engravings interpreted in the specific textual passages. The photographs in *World Journey* are published on thirty-two glossy plates, grouped together in five separate sections. The order in which the photographs of phallic artefacts appear generally corresponds to the order of the textual discussion of the depicted objects. In both volumes, the textual passages that accompany the images promise to elucidate the meaning of the material objects.

Throughout their volumes, Knight and Hirschfeld use the textual framework to draw attention to the fact that there is no easy access to or simple consensus concerning the meaning of phallic artefacts, which can be interpreted in different ways.^49^ This uncertainty is amplified by the fact that the material objects belong to the past and often originate in unfamiliar cultural contexts, which raises further questions about their original meaning. As such, the textual framework also highlights the difficulties involved in making sense of the illustrations depicting these objects. Both authors suggest that the images are open to different interpretations and therefore require careful textual framing and scholarly explication. In turn, Knight and Hirschfeld take on the position of expert interpreters who can make sense of the artefacts and their visual representations and explain their significance to readers. Moreover, they suggest that the restrictive forces at work in Western culture make it difficult to understand the genuine meaning of these objects, since they are all too easily dismissed as obscene. It is by offering scholarly readings of object and image that both authors secure their scientific authority and present themselves as radical thinkers capable of producing a form of sexual knowledge that can challenge allegedly restrictive Western attitudes towards sex.

The challenges involved in interpreting the material remains of phallic worship are reiterated throughout *Worship of Priapus*. Knight concedes that objects might seem offensive or obscene when taken out of their historical and cultural context, but maintains that these artefacts have a different *‘hidden meaning’* that becomes apparent when considering their original historical period and culture of production.^50^ The frontispiece of the book (figure 1) depicts four wax phalluses acquired by fellow Dilettante Sir William Hamilton (1730–1803) from a church in Isernia, Italy in 1780. Hamilton donated the objects to the British Museum in 1784, where Knight’s engraver produced the illustration in 1786.^51^ In the accompanying letter, dated 1781, Hamilton explains that the phalluses were sold as votive offerings, and speculates about their ritualistic function in, for instance, female fertility rites.^52^ Based on his observations, he suggests that these sexual objects have a significance within eighteenth-century rural Catholic culture that is obscured when they are dismissed as obscene. Similarly, *Worship of Priapus* includes illustrations of sexually explicit ancient Roman amulets (figure 2), some of which had also been collected by Hamilton and bequeathed to the British Museum. Knight explains that these amulets depicting female and male sexual organs were not seen as obscene by the ancients, but were revered and ‘worn by devout persons of antiquity’, as they ‘represented the act of generation which was considered as a solemn sacrament, in honour of the Creator’.^53^ Here and elsewhere Knight presents images that show sexually explicit objects, but then questions the idea that these artefacts are obscene, pointing instead to their ritualistic and religious (rather than merely sexual) significance.

In discussions of other engravings, Knight draws attention to the sexual nature of the illustration only to reveal the deeper symbolic and religious significance of the artefacts. The illustration of one of the erotic carvings in the Elephanta Caves (Gharapurichi Leni) near Mumbai, in India (figure 3), for instance, shows a man performing oral sex on a woman. In the accompanying text, Knight explains that cunnilingus and fellatio had a religious meaning in Hinduism, standing as ‘a symbol of refreshment and invigoration’.^54^ Another explicit image (figure 4) shows a statue of the Greco-Roman god Pan having sex with a female goat. At the time, this object was in the private collection of Knight’s friend Charles Townley. Knight had it engraved especially for the publication, as he states, ‘for the benefit of the learned’.^55^ He discusses this piece along with the more famous statue of the same subject, which was first discovered during excavations of Herculaneum in 1752, and was later introduced to the Naples museum’s ‘Secret Cabinet’.^56^ In letters written before the publication of *Worship of Priapus*, the Pan and Goat illustration was highlighted by Knight as his primary concern if the book were to be made ‘in the smallest degree public’.^57^ Still, he maintains that even the act depicted in this piece—‘however shocking it may appear to the modern manners and opinions’—can and should be interpreted as sacred. He explains that the sexual union of satyr and animal in antiquity ‘represent[s] the reciprocal incarnation of man with the deity, when incorporated with universal matter’.^58^ This pushing of the limits of propriety in the images he reproduced was central to Knight’s attempt to convince his readers that, with careful cross-cultural and cross-historical examination of objects, it was possible to uncover the unexpected symbolic and religious meaning of even the most sexually explicit material.

In the case of sexually explicit objects, Knight seeks to draw attention to the spiritual rather than merely sexual meaning of the artefact. On the other hand, he also reveals to his readers the deeper sexual significance of apparently ‘innocent’ objects. For instance, the granite statue of a bull at Thanjavur (figure 5) is explained, like the Pan and the Goat sculptures, as part of phallic worship rites that celebrate divine generative powers through animal imagery.^59^ Similarly, in his discussion of an engraving of a lotus flower (figure 6) he points out that this plant has been adopted widely as a symbol of fertility and reproduction.^60^ In these instances, the textual framework and Knight’s expert reading of the depicted objects are required to reveal a sexual dimension to the image, and to initiate the reader into the understanding of this hidden meaning.

Like Knight, Hirschfeld includes two photographs of what he describes as ‘unambiguously phallic symbols’ in Java and Japan (figures 7 and 8), which he seeks to understand in terms of their ritualistic function in their cultures of origin.^61^ Other photographs of phallic materials in *World Journey*, however, are of artefacts and everyday objects that are not sexually explicit, but have been appropriated to the context of phallic worship rites. The photograph of Hirschfeld posing with a cannon (figure 9), for instance, is accompanied by a textual description that situates the object in the context of Javanese fertility rituals. According to Hirschfeld, Javanese women worshipped objects they perceived as phallic, including rocks and trees, to increase their own fertility.^62^ The cannon, in particular, was attended by thousands of women every year who offered flowers in the hope of achieving pregnancy. It is Hirschfeld’s expert knowledge of Asian cultures that allows him to decode these images by revealing the sexual significance of the objects depicted.

Rather than presenting definitive explanations, Knight and Hirschfeld also highlight the idea that objects can take on various meanings in different historical and cultural contexts. In his discussion of artefacts seen to depict acts of bestiality, such as the Pan and the Goat statues, Knight offers a symbolic reading that goes beyond the sexual dimension of the object. At the same time, he does not reject the idea that some of these objects might depict real sexual acts between humans and animals. According to Knight, the Egyptians were among ancient cultures that did perform rituals involving a woman having sex with a goat, which was seen as ‘a representation of the incarnation of the Deity, and the communication of his creative spirit to man’.^63^ Here Knight establishes his scholarly expertise by offering a number of interpretations that are based on his knowledge of different cultural and historical contexts. In so doing, he also encourages readers to think about the contingency of sexual morals and attitudes across cultures and historical periods.^64^

**Figure 10.**
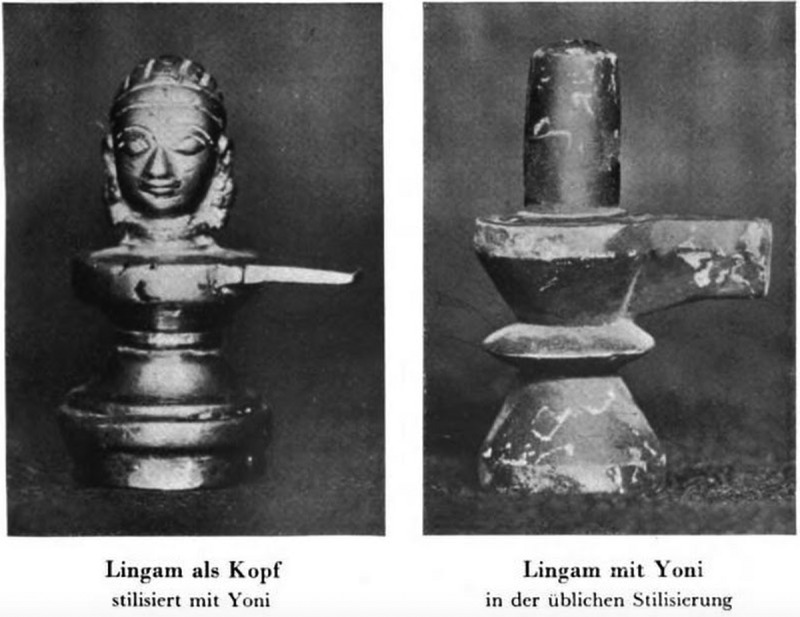
‘Lingam as head, stylised with yoni’ and ‘Lingam with yoni in the usual stylisation [India]’; from Magnus Hirschfeld, *Die Weltreise eines Sexualforschers* (Brugg: Bözberg, 1933), pl. 27.

Hirschfeld, too, puts emphasis on the ambiguity of phallic objects to demonstrate his scientific knowledge and expertise. This is particularly evident in his exploration of lingam and yoni worship in India. Accompanying the photographs of two lingam and yoni objects (figure 10)—one showing the lingam with a human head, the other in a more reduced, stylized form—is a textual passage that raises the question of how such objects might be received in early twentieth-century Indian culture. Drawing on the opinions of Indian scholars, Hirschfeld offers three explanations: first, that women are not aware of the fact that the lingam represents a phallus and that it has sexual connotations; second, that the lingam is obviously phallic and that even children are aware of its sexual meaning; and third—citing his friend, the South Asian psychoanalytic writer Girindrasekhar Bose (1887–1953)—that knowledge about the sexual connotations of the lingam is located on the level of the ‘Unterbewußte’ (subconscious).^65^ Hirschfeld does not offer a definitive statement concerning the reception of phallic worship in India, but rather displays his authority by offering an informed discussion of the difficulties involved in determining how these objects have been understood and received in the past and present. Here, as in the other examples discussed in this section, the textual framework highlights the ambiguity of the phallic objects and their visual representations to affirm the expert knowledge and scientific authority required to grasp the meaning of these artefacts.

## Phallic objects, resistance, and the Western tradition

Knight and Hirschfeld also draw upon phallic objects and their visual representations to counter what they perceive as the development of a sexually restrictive Western culture strongly shaped by Christianity. In so doing, they take up self-consciously radical positions in relation to their contemporaries. Both authors suggest that phallic worship stands for an unrestrained acknowledgement of the importance of sex that was specific to a more liberated ‘primitive’ past. Building on and contributing to Western traditions of cultural appropriation, Knight and Hirschfeld both mobilize the concept of the primitive to work through their own ideas about sex.^66^ Their aim is to bring to light alternative attitudes to sex found in allegedly primitive cultures to enlighten and liberate Western audiences. Historical phallic objects and their illustrations are seen to manifest the primitive past in the present and can therefore be used to oppose the restrictive forces associated with Western civilization and Christian religion.^67^

Knight’s engagement with phallic worship serves to express his anticlerical views. For instance, the phallic rites discovered by Hamilton in contemporary Italy are presented as remnants of the primitive pagan worship of sexual power. They are seen to demonstrate not only the ‘similitude of the Popish and pagan religion’ but also that Catholicism, for all its attempts to deny the importance of sexual pleasure, was directly descended from this primal veneration of sex.^68^ For Knight, this revealed the hypocrisy of Christian religion, which had contributed to society’s alienation from what he perceived to be a more natural experience of sex. He sought to open up access to this past through the study of the material remains of phallic worship.

While Knight positioned himself within a small clique of like-minded free thinkers, and never attempted to circulate his ideas beyond this restricted circle, Hirschfeld reached out to a wider audience and used his scientific authority to advocate for social and legal change. In *World Journey*, he turns to Asian phallic worship traditions to manufacture a sexually unrestrained past that will allow him and his intended Western audience to challenge sexual attitudes in the Western world. Although Hirschfeld was critical of hierarchical evolutionary narratives that condemned non-Western cultures as inferior and uncivilized, his construction of the primitive past in his travel narrative is clearly shaped by the ideologies of late nineteenth- and early twentieth-century colonialism.^69^

It is particularly striking that Hirschfeld fails to mention Western phallic worship and does not acknowledge scholarship by writers like Knight, who had studied phallic rites for 150 years and whose central tenets were clearly influential on his own.^70^ Indeed, it is evident that Hirschfeld is familiar with the ancient Roman material, since *World Journey* includes a single obscure reference to a Roman loaf of bread that is not explicitly sexual to the modern eye but which can be seen to have sexual significance within its ancient context.^71^ Hirschfeld thus strategically ignores Western phallic worship to locate phallic primitivism solely in Asian cultures. This erasure of phallic traditions in the Western world allows Hirschfeld to make a stronger argument about the way in which Western civilization and modern religion have restricted human sexual behaviour. His suggestion is that phallic worship was never able to thrive in the Western world and can only be located in exotic and remote Asian cultures. In so doing, he creates a greater need for the alternative understandings of sex that his scholarship promises to deliver. This allows Hirschfeld to position himself as a world traveller who has brought home an unfamiliar, radical, and liberating form of sexual knowledge.

The engagement with phallic objects in *Worship of Priapus* and *World Journey* supports these narratives about the restrictive treatment of sex in the Western world. In their different ways, Knight and Hirschfeld highlight the notion that phallic remains were subjected to censorship in the past and continue to be suppressed in the present. Knight emphasizes that it is only by good luck, and because the censors did not understand their phallic significance, that the objects he discusses have ‘escaped the attentions of the reformers’ and survived.^72^ Both authors maintain that it is because of religiously motivated censorship that such artefacts can only be found in remote and supposedly primitive spaces that have remained untouched by civilization. As has been shown, Knight’s evidence comes from the discovery of phallic rites in rural communities in southern Italy. According to Hirschfeld, phallic stones have mainly survived in parts of Asia that are distant from larger cities and thus less affected by missionary influences under Western colonial rule.^73^ This discussion of the historical treatment of phallic objects and their vulnerability to censorship allows both authors to draw attention to the restrictive forces fuelled by Christian religion and Western civilization.

Both Knight and Hirschfeld also suggest that the difficulties involved in decoding the meaning of phallic objects result from these forces of erasure and censorship. Throughout their studies, they each emphasize that the original significance of phallic artefacts, which was still understood in the primitive past, has been forgotten as cultures have become more civilized over time. With regard to Roman phallic objects, Knight explains that ‘avarice and superstition have continued these symbolical representations for ages after their original meaning has been lost and forgotten’.^74^ He further suggests that people who perform phallic rites in the present and produce phallic objects, for example in Italy or India, might be ignorant of the origins of phallic worship.^75^ Similarly, as has been argued, Hirschfeld questions the extent to which Indians understand the sexual significance of lingam and yoni objects in the present. The explanation that such knowledge might not always be fully conscious points to the links between social and psychic repression: in a cultural context in which sex is subject to censorship, the individual might not be able consciously to remember the meaning of phallic objects, although this sexual knowledge might have survived on an unconscious level.^76^

In this sense, the study of phallic worship not only serves to highlight the restrictive impact of Western civilization, but it also points to the limitations of attempts to erase sex. Knight and Hirschfeld believed that phallic worship cannot ultimately be rooted out of human cultures, because it appeals to what they understood to be universal natural sexual and reproductive desires that will persist throughout history. The surviving ancient phallic artefacts and their modern reproductions act as an important material reminder of this perseverance of phallic cults. Additionally, material phallic artefacts are invested with a specific authority: they are seen to offer more unmediated access to the past than, for instance, written sources, as they are less vulnerable to corruption.^77^ This perceived quality of material objects gave phallic artefacts a particular significance in allowing Knight and Hirschfeld to rediscover the origins of phallic worship through their scholarship.

Knight and Hirschfeld draw on this perceived power of phallic objects to materialize the past through their illustrations. Eighteenth- and nineteenth-century antiquarian scholars frequently commissioned engravers to record material objects and historical sites. In addition to offering empirical evidence, as discussed above, these images were specifically used to offer access to the past and to capture, circulate, and preserve historical knowledge.^78^ From the mid-nineteenth century onwards, photography was employed to record antiquities and ancient ruins, and it emerged as an important tool in the production and conservation of knowledge about the past.^79^ Thus engravings and photographs of phallic objects could allow Knight and Hirschfeld to construct and offer access to a primitive and sexually unrestrained primitive past.

The very act of making historical phallic artefacts available through visual representations could be seen as an expression of resistance. As has been shown, Knight was keenly aware of the power of erotic illustrations, such as the engraving of the Pan and the Goat statue, to offend contemporary tastes. It was precisely because wider audiences would deem these images offensive that they were so valuable to Knight. By virtue of their explicit nature these illustrations were seen to offer insights into cultures of the past that had been open and free enough to produce and value such artefacts. Indeed, Knight suggests that the illustrations of sexually explicit historical objects offer information that could not be conveyed textually, and this was another reason why all editions of *Worship of Priapus* had to include illustrations. For instance, he states (somewhat tongue in cheek) that he ‘shall not venture to describe’ the engraving of the carving at Elephanta depicting cunnilingus, thus highlighting that the illustration is more explicit than his textual description can afford to be.^80^ In so doing, Knight draws attention to the subversive quality of image and object, which resist the rules of decorum and decency that prevent him from expressing in writing what can be depicted visually.

Knight’s suggestion that illustrations can convey a richer sense of meaning than textual descriptions echoes deeply entrenched cultural views according to which visual images are more transparent, immediate, and natural than written texts. This assumption had particular implications with regard to sexually explicit images. Since even illiterate viewers would be able to understand such images, there were particular anxieties surrounding the publication and circulation of visual sexual materials.^81^ While the illustrations in *Worship of Priapus* were aimed at a limited and highly educated audience, Knight plays on these anxieties to emphasize the transgressive quality of his illustrations.

Although illustrations operate differently in *World Journey*, they fulfil an equally important role in enabling Hirschfeld to expose and challenge the way in which modern Western society restrained sex. In contrast to Knight’s engravings, Hirschfeld’s photographs do not show explicit sex acts or naturalistic phallic objects. The subversive force of his images derives from the suggestion that they bring home to a Western readership knowledge about phallic worship traditions that, according to Hirschfeld, are unfamiliar to a Western audience. As such, the photographs perform a function that is typical of colonial photography in that they promise to offer access to artefacts and cultures that are allegedly ‘untouched’ by the civilizing forces of the modern Western world.^82^ For Hirschfeld, these photographs offered a means to counter and resist restrictive views of sex by “making present” primitive phallic artefacts to modern Western readers.

The striking image of Hirschfeld posing between two phallic stones in Java (figure 8) in particular, stages the clash between the primitive and the modern in a way that is characteristic of colonial photography.^83^ Wearing the white suit of the colonial traveller, Hirschfeld stands for the time of modernity and progress; his figure is set into sharp relief with the primitive phallic objects he touches. The image represents the desire to engage with phallic artefacts to access the primitive past and gain historical knowledge. However, the photograph also undermines the idea that material objects and their visual representations can ever offer immediate access to the past. With Hirschfeld taking centre stage and confidently presenting the phallic stones to the viewer, the image draws attention to the ways in which knowledge about ancient objects and the past they are said to represent is always mediated through a particular lens and used for a specific set of purposes, in this case, the colonially shaped project of Western sexual science.

## Conclusions

Through its close examination of the multimedial dimensions of *Worship of Priapus* and *World Journey*, this article has demonstrated that an engagement with material culture and interest in visual evidence connect eighteenth-century antiquarianism and early twentieth-century sexual science. While acknowledging the different intellectual and social contexts that shaped their works, it has shown that Knight and Hirschfeld both turned to material objects to understand sex from cross-cultural and cross-historical perspectives. This comparative approach was central to their understanding of what it meant to produce scientific knowledge about sex and allowed them to claim positions of authority and expertise. Moreover, both authors used phallic objects and their illustrations to highlight the historical and cultural contingency of sexual attitudes and construct visions of a sexually unrestrained primitive past that served to challenge Western sexual attitudes.

Exploring these previously overlooked links between Knight and Hirschfeld offers new insights into the development of Western intellectual cultures from the eighteenth century onwards. It demonstrates the far-reaching effect of antiquarian legacies, especially with regard to cross-historical and cross-cultural thinking and the comparative study of material culture. Through its specific discussion of the ways in which antiquarianism shaped the production of sexual knowledge within sexual science, this article changes understandings of the intellectual scope and origins of the sexual scientific project. While previous scholarship has traced the beginnings of sexual science back to nineteenth-century medical disciplines, this article shows that sexual scientists drew upon different forms of evidence and varied methodologies to produce sexual knowledge and secure scientific authority. Such approaches encompassed the study of material artefacts, uses of visual evidence, and an engagement with comparative cross-historical and cross-cultural methodologies, which connect sexual science with eighteenth-century antiquarianism. As such, sexual science needs to be understood as a field of knowledge with diverse intellectual roots that include, but are not limited to, nineteenth-century medicine and that reach back (at least) to the eighteenth century.

## Notes

1 – On Knight, see Michael Clarke and Nicholas Penny, eds, *The Arrogant Connoisseur: Richard Payne Knight 1751–1824* (Manchester: Manchester University Press, 1982).

2 – So far, the argument that sexual scientists engaged with a wide range of medical and non-medical evidence has been articulated primarily by considering the intersections of sexual science and literature; Heike Bauer, *English Literary Sexology: Translations of Inversion, 1860–1930* (Basingstoke, Palgrave, 2009); Vernon Rosario, *The Erotic Imagination: French Histories of Perversity* (Oxford: Oxford University Press, 1997); Anna Katharina Schaffner, *Modernism and Perversion: Sexual Deviance in Sexology and Literature, 1850–1930* (Basingstoke: Palgrave, 2011). For more on the role historical and anthropological evidence has played in sexual science, see Kate Fisher and Jana Funke, ‘British Sexual Science Beyond the Medical: Cross-Disciplinary, Cross-Historical and Cross-Cultural Translations’, in *Sexology in Translation*, ed. Heike Bauer (Philadelphia: Temple University Press, 2015), 95–114; and Kate Fisher and Jana Funke, ‘British and German Sexual Science and the Global Search for Sexual Variation’, in *Towards a Global History of Sexual Science, 1880–1950*, ed. Veronika Fuechtner, Doug Haynes, and Ryan Jones (Berkeley: University of California Press, forthcoming 2017).

3 – For more on Hirschfeld’s journey, see Jana Funke, ‘Navigating the Past: Sexuality, Race, and the Uses of the Primitive in Magnus Hirschfeld’s *The World Journey of a Sexologist*’, in *Sex, Knowledge, and Receptions of the Past*, ed. Kate Fisher and Rebecca Langlands (Oxford: Oxford University Press, 2015), 111–34; and Charlotte Wolff, *Magnus Hirschfeld: A Portrait of a Pioneer in Sexology* (London: Quartet, 1986), 333–62. On Hirschfeld and Asia, see J. E. Bauer, ‘Magnus Hirschfeld: Panhumanism and the Sexual Cultures of Asia’, *Intersections: Gender, History and Culture in the Asian Context* 14 (2006), http://intersections.anu.edu.au/issue14/bauer.html; and Veronika Fuechtner, ‘Indian, Jews, and Sex: Magnus Hirschfeld and Indian Sexology’, in *Imagining Germany Imagining Asia: Essays in Asian–German Studies*, ed. Veronika Fuechtner and Mary Rhiel (Rochester: Camden House, 2013), 111–30.

4 – In *Worship of Priapus*, Knight draws mainly on the collection of fellow antiquarian Charles Townley. On Knight and Hirschfeld as phallic collectors, see Giancarlo Carabelli, *In the Image of Priapus* (London: Duckworth, 1996); and Jennifer Grove, ‘Anglo-American Collectors of Sexually-Related Antiquities in the 19th and Early 20th Century’ (Ph.D. dissertation; University of Exeter, 2013).

5 – Bruce Redford, *Dilettanti: The Antic and the Antique in Eighteenth-Century England* (Los Angeles: Getty Museum, 2008), 115–16; 200. Knight also worked with the engraver Georg Hackert; Carabelli, *Image of Priapus*, 78. The term ‘phallic’ was used loosely by antiquarians and collectors like Knight to describe both explicitly phallic, and otherwise sexually themed, artefacts as well as other objects that were seen to have had a ritual function related to sexuality.

6 – A number of photographs show Hirschfeld lecturing or in the company of colleagues, while others depict people he met or mentions in his text. It is not known who took specific images, although Hirschfeld explains that some photographs were taken by him or by his travel companion and partner Tao Li (Li Shiu Tong). He also mentions that photographs were given to him by colleagues and friends. See Magnus Hirschfeld, *Die Weltreise eines Sexualforschers* (Frankfurt am Main: Eichborn, 2006), 78–79, 105.

7 – The number of photographs included in the American and English editions of Hirschfeld’s book, both published in 1935, as well as the placement of these images in relation to the text are different from the first German edition to which this article refers. See Magnus Hirschfeld, *Women East and West: Impressions of a Sex Expert* (London: Heinemann, 1935), and *Magnus Hirschfeld, Men and Women: The World Journey of a Sexologist* (New York: G.P. Putnam’s Sons, 1935).

8 – For example, Ivan Crozier, ‘Nineteenth-Century British Psychiatric Writing about Homosexuality before Havelock Ellis: The Missing Story’, *Journal of the History of Medicine and Allied Sciences* 63, no. 1 (2008): 65–102.

9 – For example, Ivan Crozier, ‘Pillow Talk: Credibility, Trust and the Sexological Case History’, *History of Science* 46, no. 4 (2008): 375–404; and Harry Oosterhuis, *Stepchildren of Nature: Krafft-Ebing, Psychiatry, and the Making of Sexual Identity* (Chicago: University of Chicago Press, 2000). For scholarship that has begun to explore the ways in which other forms of evidence are used within sexual science, see note 2.

10 – For example, Ian Jenkins and Kim Sloan, *Vases & Volcanoes: Sir William Hamilton and His Collection* (London: British Museum Press, 1996); and Rosemary Sweet, *Antiquaries: The Discovery of the Past in Eighteenth-Century Britain* (London: Hambledon, 2004).

11 – Jason M. Kelly, *The Society of Dilettanti: Archaeology and Identity in the British Enlightenment* (New Haven and London: Yale University Press, 2009).

12 – Richard Payne Knight to Sir Joseph Banks, 18 June 1785; Papers of Sir Joseph Banks, 1745–1923 (bulk 1745–1820), National Library of Australia. MS 9, 102b, http://nla.gov.au/nla.obj-222969158.

13 – See note 10.

14 – On the Dilettanti’s encounter with these images, see Carabelli, *Image of Priapus*, 27–28.

15 – Peter Funnell, ‘The Symbolical Language of Antiquity’, in Clarke and Penny, *Arrogant Connoisseur*, 50–64, at 52.

16 – Magnus Hirschfeld, *Weltreise eines Sexualforschers*, 27. For a discussion of Hirschfeld’s anthropological approach, and the differences between ethnology and anthropology in the German context, see Funke, ‘Navigating the Past’.

17 – The relation between anthropology and sexual science has begun to be explored, for instance, by Andrew P. Lyons and Harriet Lyons, *Irregular Connections: A History of Anthropology and Sexuality* (Lincoln: University of Nebraska Press, 2004). See also notes 2 and 3.

18 – For a discussion of Hirschfeld’s critical stance towards religion, see Elena Mancini, *Magnus Hirschfeld and the Quest for Sexual Freedom: A History of the First International Sexual Freedom Movement* (New York: Palgrave, 2010), 12–13.

19 – According to Whitney Davis, Knight believed that this generative force found its origin in ‘an abstract if archaic principle of pansexual attraction […] whether sexually active or passive, anatomically male or female, or erotically homo-, bi-, or heterosexual’; Whitney Davis, *Queer Beauty: Sexuality and Aesthetics from Winckelmann to Freud and Beyond* (New York: Columbia University Press, 2010), 72.

20 – Research in this area is limited, especially with regards to early twentieth-century interest in phallic worship, but see Grove, ‘Anglo-American Collectors’.

21 – For example, Hodder Michael Westropp, C. Staniland Wake, and Alexander Wilder, *Ancient Symbol Worship: Influence of the Phallic Idea in the Religions of Antiquity* (London: privately published, 1874). See Lyons and Lyons, *Irregular Connections*, 57.

22 – On India, see Edward Sellon, ‘On the Phallic Worship of India’, in *Memoirs Read Before the Anthropological Society of London* (London: Trübner & Co., 1863–64), 327–34. On Africa, see Sir Richard F. Burton, ‘Notes on Certain Matters Connected with the Dahoman’, *Memoirs Read Before the Anthropological Society of London* (London: Trübner & Co., 1863–64), 308–21.

23 – *Memoirs Read Before the Anthropological Society of London* (London: Trübner & Co., 1865), 273–74.

24 – Carabelli, *Image of Priapus*, 111–12.

25 – Catherine Johns, *Sex or Symbol: Erotic Images of Greece and Rome* (London: British Museum, 1982), 28–29.

26 – Sigmund Freud and Ivan Bloch, for instance, whose work Hirschfeld had read, engaged explicitly with Knight and later studies of phallic worship. Hirschfeld’s interest in phallic cults has received limited scholarly attention, but see Bauer ‘Magnus Hirschfeld’, par. 17–18; Funke, ‘Navigating the Past’; and Grove, ‘Anglo-American Collectors’.

27 – Carabelli, *Image of Priapus*, 7–11; Noah Heringman, *Sciences of Antiquity: Romantic Antiquarianism, Natural History, and Knowledge Work* (Oxford: Oxford University Press, 2013); Alain Schnapp, ‘Antiquarian Studies in Naples at the End of the Eighteenth Century: From Comparative Archaeology to Comparative Religion’, in *Naples in the Eighteenth Century: The Birth and Death of a Nation State*, ed. Girolamo Imbruglia (Cambridge: Cambridge University Press, 2000), 154–66.

28 – The claim of Richard Colt Hoare that antiquarians ‘speak from facts not theory’ is much quoted; Richard Colt Hoare, *The Ancient History of South* [*and North*] *Wiltshire* (London: W. Miller, 1812), 7. See also Sweet, *Antiquaries*, 345–50.

29 – Arnaldo Momigliano, *Studies in Historiography* (London: Weidenfeld & Nicholson, 1966).

30 –Susan Marchand, ‘From Antiquarian to Archaeologist? Adolf Furtwängler and the Problem of Modern Classical Archaeology’, in *Momigliano and Antiquarianism: Foundations of the Modern Cultural Sciences*, ed. Peter N. Miller (Toronto: University of Toronto Press, 2007), 248–85.

31 – Hayden White, ‘The Politics of Historical Interpretation: Discipline and De-Sublimation’, *Critical Inquiry* 9, no. 1 (1982): 113–37.

32 – See note 27.

33 – On Knight’s anti-clericalism, see G. S. Rousseau, ‘The Sorrows of Priapus: Anticlericalism, Homosocial Desire, and Richard Payne Knight’, in *Sexual Underworlds of the Enlightenment*, ed. Roy Porter and G. S. Rousseau (Chapel Hill: University of North Carolina Press, 1988), 101–53.

34 – Richard Payne Knight, *An Account of the Remains of the Worship of Priapus* (London: T. Spilsbury, 1786), 23–24.

35 – Ibid., 24.

36 – James D. Steakley, ‘Per scientiam ad justitiam: Magnus Hirschfeld and the Sexual Politics of Innate Homosexuality’, in *Science and Homosexualities*, ed. Vernon A. Rosario (New York: Routledge, 1997), 133–54.

37 – Mancini, *Magnus Hirschfeld*, 61–70.

38 – On this general point, see W. J. T. Mitchell, *Iconology: Image, Text, Ideology* (Chicago: University of Chicago Press, 1987), 37–40, 152.

39 – Stephanie Moser and Sam Smiles, *Envisioning the Past: Archaeology and the Image* (Malden: Blackwell, 2005), 4–5; Sam Smiles, *Eye Witness: Artists and Visual Documentation in Britain, 1770–1830* (Aldershot: Ashgate, 2000).

40 – Carabelli, *Image of Priapus*, 128.

41 – Richard Payne Knight to Sir Joseph Banks, 18 June 1785; Papers of Sir Joseph Banks, 1745–1923 (bulk 1745–1820), National Library of Australia. MS 9, 102b, http://nla.gov.au/nla.obj-222969158.

42 – For more on the typological approach in antiquarianism, see Susan Pearce, ‘Antiquaries and the Interpretation of Ancient Objects, 1770–1820’, in *Visions of Antiquity: The Society of Antiquaries in London, 1707–2007*, ed. Susan Pearce (London: Society of Antiquaries, 2007), 147–72, at 158–59.

43 – Christopher Evans, ‘“Delineating Objects”: Nineteenth-Century Antiquarian Culture and the Project of Archaeology’, in Pearce, *Visions of Antiquity*, 267–305, at 274–75; Sam Smiles, ‘Art and Antiquity in the Long Nineteenth Century’, in Pearce, *Visions of Antiquity*, 123–45, at 124.

44 – Ann Thomas, *Beauty of Another Order: Photography in Science* (New Haven and London: Yale University Press, 1997); Lorraine Daston and Peter Galison, ‘The Image of Objectivity’, *Representations* 40 (1992): 81–128; Christopher Pinney, *Photography and Anthropology* (London: Reaktion, 2011).

45 – For more on Hirschfeld and medical photography, see David James Prickett, ‘Magnus Hirschfeld and the Photographic (Re)Invention of the “Third Sex”’, in *Visual Culture in Twentieth-Century Germany: Text as Spectacle*, ed. Gail Finney (Bloomington: Indiana University Press, 2006), 103–19. Hirschfeld’s use of photography beyond his medical publications has not yet received any scholarly attention.

46 – Pinney, *Photography and Anthropology*.

47 – The photographs are arranged differently in the various existing editions of *World Journey*. The discussion here is based on the first German edition of the book: Magnus Hirschfeld, *Weltreise eines Sexualforschers* (Brugg: Bözberg, 1933).

48 – Moser and Smiles, *Envisioning the Past*, 5–6.

49 – For more on objects and ambiguity, see Sven Dupré and Christoph Lüthy, eds, *Silent Messengers: The Circulation of Material Objects of Knowledge in the Early Modern Low Countries* (Berlin: Lit, 2011); and Ian Hodder, ed., *The Meaning of Things: Material Culture and Symbolic Expression* (London: Unwin Hyman, 1989).

50 – Knight, *Worship of Priapus*, 24 (original emphasis).

51 – Davis, *Queer Beauty*, 55.

52 – Carabelli, *Image of Priapus*, 12–14.

53 – Knight, *Worship of Priapus*, 47.

54 – Ibid., 81.

55 – Ibid., 56.

56 – Ibid. See Kate Fisher and Rebecca Langlands, ‘Bestiality in the Bay of Naples: The Herculaneum Pan and Goat Statue’, in Fisher and Langlands, *Sex, Knowledge, and Receptions of the Past*, 86–110.

57 – Richard Payne Knight to Sir Joseph Banks, 18 June 1785; Papers of Sir Joseph Banks, 1745–1923 (bulk 1745–1820), National Library of Australia. MS 9, 102b, http://nla.gov.au/nla.obj-222969158.

58 – Knight, *Worship of Priapus*, 56, 59.

59 – Ibid., 57.

60 – Ibid., 84–86.

61 – Hirschfeld, *Weltreise*, 210. The translation is the authors’ own.

62 – Ibid., 192.

63 – Knight, *Worship of Priapus*, 55.

64 – Ibid.

65 – Hirschfeld, *Weltreise*, 283–84; Freud preferred the term ‘Unbewußte’ (unconscious), but imprecise usage of psychoanalytic terminology was common even among authors familiar with psychoanalytic ideas.

66 – Elazar Barkan and Ronald Bush, eds, *Prehistories of the Future: the Primitivist Project and the Culture of Modernism* (Stanford: Stanford University Press, 1995); Johannes Fabian, *Time and the Other: How Anthropology Makes Its Object* (New York: Columbia University Press, 1983); Lyons and Lyons, *Irregular Connections*, 100–30.

67 – For more on historical objects representing the past in the present, see Pearce, ‘Antiquaries and the Interpretation of Ancient Objects’, 161–66. For a discussion of the role of illustrations in reproducing the past, see Smiles, *Eye Witness*, 9; and Heringman, *Sciences of Antiquity*. For more on material objects in the context specifically of sexual knowledge about the past, see Kate Fisher and Rebecca Langlands, ‘“This Way to the Red Light District”: The Internet Generation Visits the Brothels in Pompeii’, in *Classics For All: Reworking Antiquity in Mass Culture*, ed. Dunstan Lowe and Kim Shahabudin (Newcastle Upon Tyne: Cambridge Scholars, 2009), 172–94; and Fisher and Langlands, ‘Bestiality’.

68 – Knight, *Worship of Priapus*, 4.

69 – For more on Hirschfeld and the primitive, see Funke, ‘Navigating the Past’.

70 – Ibid., 130–33. See note 26.

71 – Hirschfeld, *Weltreise*, 284.

72 – Knight, *Worship of Priapus*, 47.

73 – For instance, Hirschfeld, *Weltreise*, 79, 210.

74 – Knight, *Worship of Priapus*, 24.

75 – Ibid., 81–82.

76 – Davis, *Queer Beauty*, 51–81.

77 – Fisher and Langlands, ‘Red Light District’; Fisher and Langlands, ‘Bestiality’.

78 – Kelly Eileen Battles, ‘The Antiquarian Impulse: History, Affect, and Material Culture in Eighteenth- and Nineteenth-Century British Literature’ (Ph.D. dissertation; Michigan State University, 2008), 121–64; Frederick N. Bohrer, ‘Photography and Archaeology: The Image as Object’, in Moser and Smiles, *Envisioning the Past*, 180–91.

79 – Jennifer Green-Lewis, *Framing the Victorians: Photography and the Culture of Realism* (Ithaca: Cornell University Press, 1996); Claire Lyons, *Antiquity and Photography: Early Views of Ancient Mediterranean Sites* (Los Angeles: Paul Getty Museum, 2005).

80 – Knight, *Worship of Priapus*, 81.

81 – For more on sexually explicit illustrations and audience, see Lisa Z. Sigel, *Governing Pleasures: Pornography and Social Change in England, 1815–1914* (New Brunswick: Rutgers University Press, 2002), 23–27.

82 – Anne Maxwell, *Colonial Photography and Exhibitions: Representations of the* ‬*‘Native’ and the Making of*‬‬‬‬‬‬‬‬‬‬‬‬‬‬‬‬‬‬‬ *European Identities* (London: Leicester University Press, 1999), 10.

83 – Anne McClintock, *Imperial Leather: Race, Gender, and Sexuality in the Colonial Contest* (New York: Routledge, 1995), 125.

